# Risk Factors Associated with Benign and Malignant Thyroid Nodules in Autoimmune Thyroid Diseases

**DOI:** 10.1155/2013/673146

**Published:** 2013-05-25

**Authors:** Priscila Carneiro Moreira Lima, Arnaldo Moura Neto, Marcos Antonio Tambascia, Denise Engelbrecht Zantut Wittmann

**Affiliations:** Endocrinology Division, Internal Medicine Department, Faculty of Medical Sciences, University of Campinas, Rua Tessalia Vieira de Camargo 126, Barao Geraldo, Campinas 13084-971, SP, Brazil

## Abstract

*Objectives*. Assess the prevalence of thyroid nodules and predictors of malignant origin in patients with autoimmune thyroid diseases. *Patients and Methods*. Retrospective study including 275 patients, 198 with Graves' disease and 77 with Hashimoto's thyroiditis. Clinical and demographical data, ultrasonographical nodule characteristics, total thyroid volume and histological characteristics were recorded. *Results*. Graves' disease: the prevalence of thyroid nodules and thyroid carcinoma were 27.78% and 5.05%, respectively. Older age (OR = 1.054; 95% CI = 1.029–1.080) and larger thyroid volumes (OR = 1.013; 95% CI = 1.003–1.022) increased the chance of nodules. Younger age (OR = 1.073; 95% CI = 1.020–1.128) and larger thyroid volume (OR = 1.018; 95% CI = 1.005–1.030) predicted thyroid carcinoma. Hashimoto's thyroiditis: the prevalence of thyroid nodules and carcinomas were 50.7% and 7.8%, respectively. Nodules were predicted by thyroid volume (OR = 1.030; 95% CI = 1.001–1.062). We found higher number of nodules in patients with thyroid carcinoma than in those with benign nodules (3 versus 2; *P* = 0.03). Patients with Hashimoto's thyroiditis presented nodules more frequently than patients with Graves' disease (50.65% versus 27.28%; *P* < 0.001), while the prevalence of carcinoma was similar (*P* = 0.751). *Conclusions*. Larger goiter was associated with carcinoma in Graves' disease and Hashimoto's thyroiditis. Younger patients presented higher risk of papillary thyroid carcinoma in Graves' disease. The prevalence of carcinoma was similar in both conditions.

## 1. Introduction

Association between thyroid autoimmunity and nodules or carcinoma has been suggested in many previous works, but its clinical significance is still uncertain [1]. Whether all patients with autoimmune thyroid diseases are at increased risk for nodules and thyroid cancer or if there are certain individual characteristics that augment this risk is still debatable. 

In patients with Graves' disease (GD), nodules are detected in 10–31% of cases [2], and approximately half will present nodules during followup [3]. Some studies have shown that around 17% of those nodules are malignant, whereas in the healthy population this estimate is 5% [4]. It is also known that 1.7–2.5% of patients with GD present malignant nodules, compared to 0.25% in the general population [5], evidencing a greater risk for thyroid carcinoma in this condition. There is a strong link between thyroid carcinoma and GD, which can be considered an adverse prognostic factor [6]. 

Hashimoto's thyroiditis (HT) is frequently associated with small thyroid nodules, resulting from the autoimmune process. Ultrasound (US) is essential in differentiating true nodules from pseudonodules, which are an expression of the inflammatory infiltrate. Previous works suggest a higher frequency of thyroid carcinoma in these patients, but this is not unanimous [7]. Some authors believe that the augmented immune response could be associated with carcinogenesis [8], despite the good prognosis found in patients with papillary thyroid carcinoma (PTC) and HT [9]. 

There is a paucity of data regarding the study of clinical and laboratorial characteristics as well as pathology and evolution of this association between thyroid autoimmunity and cancer. We studied patients with both autoimmune thyroid diseases in order to assess the variables associated with the presence of thyroid nodules and identify those related to thyroid carcinoma.

## 2. Patients and Methods

### 2.1. Study Design

This was a retrospective study of 275 patients, 198 with Graves' disease and 77 patients with HT. Data were collected from patients' charts. All were followed in the Outpatient Clinic for Thyroid Function Disorders at the Endocrinology Division, Hospital das Clinicas, University of Campinas, from May 2009 to April 2010. All patients were from an iodine sufficient area.

The diagnosis of GD was based on clinical history and signs of hyperthyroidism, elevated serum-free thyroxine (FT4) concentration as well as suppressed TSH levels, the presence of thyroid antibodies (antithyroperoxidase (TPOAb) and/or antithyroglobulin (TGAb)), and 99mTc-pertechnetate uptake results. Disease was considered active if serum TSH levels at the time of data collection were below the reference range or if the patient still required antithyroid medication for maintenance of euthyroidism. Patients were considered to be in remission if euthyroid without anti-thyroid medication for at least 6 months or if on L-T4 replacement after definitive treatment.

HT diagnosis was based on clinical history and signs of hypothyroidism, elevated TSH levels, and/or reduced concentrations of FT4, associated with elevated TPOAb and TGAb levels. 

Patients with ultrasonographically diagnosed thyroid nodules were submitted to FNAB and cytological examination. Patients with cytology diagnostic or suspicious for malignancy were referred for thyroidectomy and histopathological confirmation. Histological characteristics of thyroid carcinoma were studied for GD and HT separately, and concurrently with the following variables: age, gender, time of hyper/hypothyroidism diagnosis, age at diagnosis, smoking habit, disease activity, treatment duration, and ultrasound (parenchyma and nodule characteristics, thyroid volume).

The University's Ethics in Research Committee approved the study. The present work was done in accordance with the Declaration of Helsinki (British Medical Journal, 1964, ii, 177).

### 2.2. Biochemical Analysis

TSH was measured by electrochemiluminescence assay (Elecsys TSH immunoassay analyzer-Roche)—reference value (RV) 0.4–4.5 mUI/L. FT4, TPOAb, and TGAb levels were determined by electrochemiluminescence in the same analyzer (RV: FT4: 0.9–1.8 ng/mL; TPOAb: <35 *μ*UI/mL and TGAb: <115 *μ*UI/mL).

### 2.3. Ultrasound

Data from ultrasound evaluations were collected from chart review, with thyroid descriptive-morphological analysis including lobe dimensions, parenchymal echogenicity, and nodule characteristics. All exams were done in the Radiology Department of our University Hospital. Total thyroid volume was calculated from the ellipsoid formula [10], adding the volumes of the left and right lobes and isthmus. 

### 2.4. Thyroid 99mTc-Pertechnetate Uptake

In the 15 days preceding the exam, patients were instructed to abstain from iodine rich food and personal care products. Twenty minutes after intravenous injection of 10mCi (370MBq) of 99mTc-pertechnetate, thyroid uptake was read. Data regarding scintigraphy uptake was collected from patients' records. Thyroid 99mTc pertechnetate uptake scan was performed in the evaluation of patients with thyroid nodules and serum thyrotropin (TSH) values below the normal range in order to identify nonfunctioning nodules, which were directed to FNAB.

### 2.5. Statistical Analysis

Descriptive analysis of data was done by the elaboration of frequency tables for categorical variables and measurement of position and dispersion for numerical variables. The association between categorical variables was verified by the chi-square test or Fisher's exact test, as appropriate. Comparison between measurements of two groups was obtained by the Mann-Whitney test and between 3 groups by the Kruskal-Wallis test. 

Identification of factors that influence the presence of benign thyroid nodules and thyroid carcinoma was done by univariate and multivariate logistic regression analysis.

Data are reported as medians (range) or frequency (percentage) unless otherwise stated. The significance level adopted was 5% (*P* < 0.05) for all tests. All calculations were done using the Statistical Analysis System (SAS) for Windows (SAS Institute Inc, 2002–2008, Cary, NC, USA), version 9.2.

## 3. Results

### 3.1. Patients with Graves' Disease

Demographic characteristics of patients with GD are shown in Table 1. Data were missing for 65 patients in smoking habit and for 61 regarding the presence of ophthalmopathy (not discriminated in the patients' charts). 

 The prevalence of thyroid nodules and thyroid carcinoma was 27.78% (55/198) and 5.05% (10/198), respectively. Radioiodine was the treatment of choice for hyperthyroidism for 66 (33.4%) patients and 17 (25.8%) of these presented nodules, none malignant.

### 3.2. Thyroid Nodules and Graves' Disease

Among the 55 patients (51 female and 1 male) with thyroid nodules, 46 (83.6%) had benign disease and 9 (16.4%) malignant. Fifty (90.9%) patients presented active disease, and 17 (30.9%) had undergone radioiodine treatment. Median number of nodules was 2 (1–7), and median size (largest diameter of three dimensions) was 1.7 cm (0.5–6.8 cm). Mean time of followup was 7.5 and 5.8 years for those with and without thyroid nodules, respectively. Median thyroid volume was 23.1 cc (1.6–201.3 cc) in patients not presenting nodules on US and 29.8 cc (4.1–242 cc) for those with nodules (*P* = 0.019).

Assessment of predictors for thyroid nodule development by univariate logistic regression analysis revealed that older age (OR = 1.054, 95% CI = 1.031–1.079; *P* < 0.001), older age at diagnosis (OR = 1.047, 95% CI = 1.024–1.071; *P* < 0.001), longer disease duration (OR = 1.063, 95% CI = 1.003–1.128; *P* = 0.04), and larger total thyroid volume (OR = 1.012, 95% CI = 1.004–1.021; *P* = 0.004) as significant risk factors. The multivariate model adjusted for all variables showed that older age (OR = 1.054, 95% CI = 1.029–1.080; *P* < 0.001), and larger total thyroid volume (OR = 1.013; 95% CI = 1.003–1.022; *P* = 0.007) were the independent predictors for thyroid nodules in the patients with GD—Table 2.

When excluding patients with thyroid carcinoma, older age (OR = 1.067, 95% CI 1.040–1.095; *P* < 0.001), older age at diagnosis (OR = 1.061, 95% CI = 1.035–1.088; *P* < 0.001) and larger total thyroid volume (OR = 1.011, 95% CI = 1.002–1.020; *P* = 0.017) were predictors for development of benign nodules. 

### 3.3. Thyroid Carcinoma and Graves' Disease

In the 10 patients presenting thyroid carcinoma, 9 were female and 1 male. Additionally, 6 were smokers, 5 patients had a history of ophthalmopathy, and all 10 had active disease. All carcinomas were PTC. Three patients had microcarcinoma. Four were classified as classic variant and 6 as follicular variant PTC. Four patients presented multifocal disease. Five patients presented hypoechogenic nodules and 3 hyperechogenic nodules. One patient was diagnosed only after surgery, and for one patient, there was no record of US echogenicity.

There were significant differences between the groups of patients with benign and malignant nodules regarding age (55.3 versus 39.0 years, resp.; *P* < 0.001) and age at diagnosis (48 versus 30 years, resp.; *P* < 0.001). Also, between patients without nodules and those with thyroid carcinoma, we found significant differences in total thyroid volume (23.1 versus 55.1 cc, resp.; *P* = 0.022)—Table 3.

Regression analysis for identification of risk factors for thyroid carcinoma in patients with thyroid nodules revealed that younger age (OR = 1.073, 95% CI = 1.020–1.128; *P* = 0.006) and younger age at diagnosis (OR = 1.087, 95% CI = 1.029–1.148; *P* = 0.003) were the predictors for carcinoma compared to benign nodules. Larger thyroid volume (OR = 1.018, 95% CI = 1.005–1.030; *P* = 0.005) was a risk factor for carcinoma when comparing patients without nodules to those with thyroid carcinoma, but not in the comparison between benign and malignant nodules (*P* = 0.26). Disease duration did not predict the presence of thyroid carcinoma when compared to benign nodules (*P* = 0.35).

### 3.4. Hashimoto's Thyroiditis

Demographic characteristics of patients with HT are shown in Table 1. The prevalence of thyroid nodules in these patients was 50.7% (39/77) and that of thyroid carcinoma 7.8% (6/77). All six patients with thyroid cancer had PTC and evidence of a nodule on US. Among the 39 patients with nodules, 37 (95%) were female, 33 (84.6%) had benign disease, and 6 (15.4%) malignant. 

### 3.5. Thyroid Nodules and Hashimoto's Thyroiditis

Comparative analysis of patients with and without thyroid nodules revealed that only thyroid volume was significantly different (18.5 versus 9.8 cc, resp.; *P* = 0.005). Patients with benign nodules presented larger total thyroid volume than those without nodules (17.3 versus 9.8 cc; *P* = 0.013). Univariate logistic regression revealed that total thyroid volume was a significant risk factor for the presence of thyroid nodules (OR = 1.030, 95% CI = 1.001–1.062; *P* = 0.049)—Table 2. 

### 3.6. Thyroid Carcinoma and Hashimoto's Thyroiditis

Six patients with HT present thyroid carcinoma, and all of them were PTC. Of these, 1 was a microcarcinoma. Four were classified as classic variant and 2 as follicular variant PTC. All six patients were female, and one of them was an active smoker. Regarding US echogenicity, 2 were detected as hypoechogenic nodules, and 4 were isoechogenic. Patients with thyroid carcinoma presented more nodules on US than patients with benign nodules (3 versus 2; *P* = 0.037), but there was no difference on the size of the largest nodule (2.1 versus 1.6 cm; *P* = 0.234)—Table 3. 

Logistic regression analysis of HT and risk factors for thyroid carcinoma was not possible due to the small number of patients with this diagnosis.

### 3.7. Comparative Analysis of Patients with Nodules in Graves' Disease and Hashimoto's Thyroiditis

Characteristics of patients with thyroid nodules in GD and HT revealed differences in total thyroid volume (29.8 versus 18.5 cc, resp.; *P* = 0.002) and treatment duration (4 versus 6 years, resp.; *P* = 0.01). The frequency of thyroid nodules was significantly increased in patients with HT compared to GD (50.65% versus 27.28%, resp.; *P* = 0.003). Patients with GD presented more frequently without nodules on US (71.72% versus 49.35%; *P* = 0.002), whereas patients with HT had more benign nodules (42.86% versus 23.23%; *P* = 0.002)—Table 4.

### 3.8. Comparative Analysis of Patients with Thyroid Carcinoma in Graves' Disease and Hashimoto's Thyroiditis

The frequency of PTC did not differ between the two groups (*P* = 0.751). Characteristics of patients with carcinoma in GD and HT showed significant differences for age (39 versus 55 years, resp.; *P* = 0.02) and age at diagnosis (30 versus 49 years, resp.; *P* = 0.004). Thyroid carcinoma in patients with GD presented more frequently as hyperechogenic nodules (37.5% versus 0%) and more frequently as isoechogenic nodules in patients with HT (66.7% versus 0%); overall Fishers exact test *P* = 0.02. Comparative analysis of patients with GD and HT regarding nodules and PTC is summarized in Table 4.

## 4. Discussion

Our study showed that the prevalence of thyroid nodules in patients with GD was 27.8%; a value that is in line with previous works [11] and is higher when compared to the healthy population [12]. We obtained the main risk factors for developing thyroid nodules in this disease, which were older age and larger thyroid volume. 

Some factors are known to be associated with greater prevalence of thyroid nodules in patients with GD. Kim et al. [13] observed that patients with nodules had an older age than those without. Besides, patients with nodules presented a larger thyroid volume. 

The prevalence of thyroid carcinoma in GD was 5%, representing 19.5% of nodules. This finding is also in accordance with the literature data [2, 11, 14, 15]. Age and thyroid volume were also involved in the risk for PTC. In GD, younger patients showed higher risk for PTC, similar to those with larger thyroid volumes, when compared to patients presenting benign nodules.

Our prevalence of thyroid carcinoma in Graves' disease matches that reported in the literature, which varies between 0.4% and 9.8% [2, 11, 14]. The same is valid for the proportion of malignant nodules (19.5%), similar to other studies [7, 15]. The finding of younger age as a risk factor for PTC is different with most other studies in this area. Kim et al. [16] found in a series of 245 patients with GD that the prevalence of nodules increases with age, but the incidence of thyroid cancer was higher in those older than 45 years (6.7% versus 1.3%; *P* = 0.05). Age was the only significant factor predicting locally advanced disease, regardless of duration or severity of hyperthyroidism and thyroid volume. Rago et al. [17], analyzing a cohort of 3,004 patients that underwent surgery from a total of 34,120 patients with autoimmune thyroid disorders, found that the independent predictors for PTC were older age, male gender, and single nodularity. Giles et al. [18] found a similar increase in risk for patients that are older than 50 years. 

Larger thyroid volumes, although not the number of nodules, were associated with the presence of both benign and malignant nodules, highlighting the key role for US and FNAB in GD. Most studies investigated the association of thyroid carcinoma and single or multiple nodules, but we did not find one that correlated with thyroid volume as estimated by thyroid US. The majority of studies found a significant higher risk for thyroid carcinoma in GD in the presence of a single nodule [19]. Erbil et al. [20], however, showed that the incidence of carcinoma in the parenchyma outside a nodule was 67%. These findings may suggest that malignancy is not always associated with nodules per se and could explain our findings of higher PTC occurrence as thyroid volume increases.

Regarding HT, the prevalence of thyroid nodules was 50.7%, compatible with other studies, as was the prevalence of carcinoma (15.4%) [7, 21]. Larger thyroid volume was associated with benign and malignant nodules. Although several works did not find influence of the number of nodules on the development of thyroid carcinoma in these patients, we found that patients with thyroid carcinoma presented a higher number of nodules compared to those with benign nodules. Gul et al. [22] compared patients with thyroid carcinoma with and without HT. The 613 patients did not differ significantly regarding the number of nodules or echogenicity.

HT showed a higher prevalence of benign thyroid nodules than GD, but the prevalence of carcinoma was similar. In patients with PTC, there were more hyperechogenic nodules in patients with GD and more isoechogenic nodules in HT patients. Most studies show thyroid carcinoma to be more likely to present as a hypoechogenic nodule [23]. This atypical presentation of thyroid carcinoma on US has been reported previously [24]. However, there is a great diversity regarding this characteristic [22, 25]. 

All cases of thyroid carcinoma were PTC, the majority occurring in women, in the presence of nodules, especially when larger than 1 cm, and disease free on long-term followup. This data are also in consonance with the majority of the current literature in this field [7, 12].

Our study has some limitations. The smaller number of patients with HT, and consequently the small number of thyroid carcinomas in this group, prevented any analysis of prediction factors for carcinoma. This is due to the single-center design of our study. Our University Hospital is a tertiary referral center and has a majority of patients with GD under followup at any given moment. However, our frequency findings in patients with HT were similar to that reported in other studies. Additionally, the lack of a control group of patients with goiter but no autoimmune thyroid disease makes the comparison with a similar population impossible. Nevertheless, our prevalence data is in line with the majority of previously published works. Our study could also have been improved if there were data available for TSH receptor antibodies in patients with GD, but unfortunately the measurement of this variable is not routine practice in our service. We believe that this could have added significant information on the models for prediction of both benign and malignant nodules. The retrospective study design also warrants confirmation of our findings on age and thyroid volume from further prospective research.

In conclusion, larger thyroid volume was associated with the presence of thyroid nodules and carcinoma in GD and to the presence of carcinoma in HT. Age is an important risk factor in GD, older for thyroid nodules and younger for carcinoma. We believe that these results should encourage more widespread US and FNAB screening in patients with autoimmune thyroid dysfunction, especially those with larger thyroid volumes. The finding of increased risk for PTC in younger patients with GD warrants confirmation from prospective studies.

## Figures and Tables

**Table 1:** Demographical and clinical characteristics of patients with Graves' disease and Hashimoto's thyroiditis.

	Graves' disease (*n* = 198)	Hashimoto's thyroiditis (*n* = 77)
Gender—male/female (%)	18/180 (9.09/90.91)	5/72 (6.49/93.51)
Age—yrs (range)	45.5 (13–84)	51 (15–84)
Age at diagnosis—yrs (range)	36.5 (9–81)	48 (5–77)
Disease duration—yrs (range)	9.08 (0.5–27)	3 (0.1–21)
Total thyroid volume—cc (range)	55.7 (1.6–242)	15.8 (1–111.9)
Nodules present (%)	55 (27.78)	39 (50.65)
PTC (%)	10 (5.05)	6 (7.79)
Time of followup—yrs (range)	5 (0.1–20)	7 (0.1–21)
Smoking (%)^a^	67 (50.38)	14 (41.18)
Time of treatment—yrs (range)	5.5 (0.1–20)	6.6 (1–16)
Ophthalmopathy (%)^b^	27 (19.71)	NA
Active disease (%)	176 (88.89)	NA
Radioiodine treatment (%)	66 (33.33)	NA

^a^ *n* = 133 for GD and *n* = 34 for HT.

^b^ *n* = 137.

NA: not available.

**Table 2:** Comparative analysis between patients with and without thyroid nodules in Graves' disease and Hashimoto's thyroiditis.

	Graves' disease (*n* = 198)			Hashimoto's thyroiditis (*n* = 77)		
	With nodules	Without nodules	*P*	With nodules	Without nodules	*P*
Gender—male/female (%)	4/51 (2.02/25.76)	14/129 (7.07/65.15)	0.581	2/37 (2.6/48.05)	3/35 (3.9/45.45)	0.675
Age—yrs (range)	53 (27–84)	43 (13–82.8)	**<0.001**	55 (15–84)	49.5 (17–79)	0.421
Age at diagnosis—yrs (range)	46 (17–81)	36 (9–77)	**<0.001**	47 (5–70)	43.5 (14–77)	0.194
Disease duration—yrs (range)	6 (0.6–20)	5.0 (0.5–27)	0.074	7 (0.2–16)	7 (0.1–21)	0.953
Total thyroid volume—cc (range)	29.8 (4.1–242)	23.1 (1.6–201.3)	**0.019**	18.5 (1–111.9)	9.8 (2.1–69.2)	**0.005**
Time of followup—yrs (range)	6 (0.3–20)	5 (0.1–20)	0.077	7 (0.2–16)	7 (0.1–21)	0.779
Smoking (%)^a^	18 (13.53)	49 (36.84)	0.885	9 (26.47)	5 (14.71)	1.0
Time of treatment—yrs (range)	4 (0.3–17)	3 (0.1–20)	0.433	6 (1–16)	7 (1–15)	0.945
Ophthalmopathy (%)^b^	22 (16.06)	70 (51.09)	0.077	NA	NA	NA
Active disease (%)	50 (25.25)	126 (63.64)	0.575	NA	NA	NA
Radioiodine treatment (%)	17 (8.59)	49 (24.75)	0.654	NA	NA	NA
99mTc uptake—% (range)	7 (0.2–82)	10 (0.3–77)	0.138	NA	NA	NA

^a^ *n* = 133 for GD and *n* = 34 for HT.

^b^ *n* = 137 for GD.

NA: not available.

**Table 3:** Comparative analysis between patients presenting with no nodules, benign nodules and thyroid carcinoma in Graves' disease and Hashimoto's thyroiditis.

	Graves' disease (*n* = 198)			*P*	Hashimoto's thyroiditis (*n* = 77)			*P*
	PTC	Benign	No nodule		PTC	Benign	No nodule	
Gender—male/female (%)	1/9 (0.51/4.55)	3/43 (1.52/21.72)	14/128 (7.07/64.65)	0.823	0/6 (0/7.79)	2/33 (2.6/42.86)	3/33 (3.9/42.86)	1
Age—yrs (range)	39 (28–58)	55.3 (27–84)	43 (13–82.8)	**<0.001** ^ c^	55 (51–84)	55 (15–75)	49.5 (17–79)	0.556
Age at diagnosis—yrs (range)	30 (17–49)	48 (20–81)	36 (9–77)	**<0.001** ^ c^	49 (44–70)	47 (5–65)	43.5 (14–77)	0.246
Disease duration—yrs (range)	9 (3–18)	5.5 (0.6–20)	5.0 (0.5–5)	0.054	8.5 (2–14)	6.7 (0.2–16)	7 (0.1–21)	0.91
Total thyroid volume—cc (range)	55.1 (16.2–167.5)	28.9 (4.1–242)	23.1 (1.6–201.3)	**0.022** ^ d^	26.7 (6.4–111.9)	17.3 (1–74.1)	9.8 (2.1–69.2)	**0.013** ^ e^
Time of followup—yrs (range)	9 (3–18)	5 (0.3–20)	5 (0.1–20)	**0.04** ^ d^	8.5 (3–14)	6 (0.2–16)	7 (0.1–21)	0.842
Smoking (%)^a^	4 (3.01)	14 (10.53)	49 (36.84)	0.757	1 (2.94)	8 (23.53)	5 (14.71)	0.523
Time of treatment—yrs (range)	3.5 (1–6)	4 (0.3–17)	3 (0.1–20)	0.416	7 (2–14)	6 (1–16)	7 (1–15)	0.984
Number of nodules (range)	1.5 (0–5)	2 (1–7)	NA	0.641*	3 (1–5)	2 (1–6)	NA	0.037*
Size of largest nodule on US—cm (range)	2.2 (0.5–6.8)	1.6 (0.7–5.7)	NA	0.172*	2.1 (1–4.3)	1.6 (0.5–4.6)	NA	0.234*
Ophthalmopathy (%)^b^	5 (3.65)	18 (13.14)	69 (50.36)	0.125	NA	NA	NA	NA
Active disease (%)	10 (5.05)	41 (20.71)	125 (63.13)	0.507	NA	NA	NA	NA
Radioiodine treatment (%)	0	17 (8.59)	49 (24.75)	0.069	NA	NA	NA	NA
99mTc uptake—% (range)	11.9 (0.8–29)	8.1 (0.2–82)	10 (0.3–77)	0.348	NA	NA	NA	NA

^a^ *n* = 133 for GD and *n* = 34 for HT.

^b^ *n* = 137 for GD.

^c^Benign versus no nodule and benign versus PTC.

^d^No nodule versus PTC.

^e^Benign versus no nodule.

*Mann-Whitney test.

NA: not available.

**Table 4:** Comparative analysis of thyroid nodules and thyroid carcinoma between patients with Graves' disease and Hashimoto's thyroiditis.

	Graves' disease	Hashimoto's thyroiditis	*P*
Nodules (*n* = 94)			

Gender—male/female (%)	4/51 (4.26/54.26)	2/37 (2.13/39.36)	1
Age—yrs (range)	53 (27–84)	55 (15–84)	0.942
Age at diagnosis—yrs (range)	46 (17–81)	47 (5–70)	0.896
Disease duration—yrs (range)	6 (0.6–20)	7 (0.2–16)	0.911
Total thyroid volume—cc (range)	29.8 (4.1–242)	18.5 (1–111.9)	**0.002**
PTC (%)	9 (9.57)	6 (6.38)	0.898
US echogenicity—hyper/iso/hypo (%)	12/28/9 (13.95/32.56/10.47)	4/19/14 (4.65/22.09/16.28)	0.073
Time of followup—yrs (range)	6 (0.3–20)	7 (0.2–16)	0.661
Smoking (%)^a^	18 (31.58)	9 (15.79)	0.439
Time of treatment—yrs (range)	4 (0.3–17)	6 (1–16)	**0.001**
Number of nodules (range)	2 (1–7)	2 (1–6)	0.861
Size of largest nodule on US—cm (range)	1.7 (0.5–6.8)	1.6 (0.5–4.6)	0.575

PTC (*n* = 16)			

Gender—male/female (%)	1/9 (6.25/56.25)	0/6 (0/37.5)	0.95
Age—yrs (range)	39 (28–58)	55 (51–84)	**0.019**
Age at diagnosis—yrs (range)	30 (17–49)	49 (44–70)	**0.004**
Disease duration—yrs (range)	9 (3–18)	8.5 (2–14)	0.786
Total thyroid volume—cc (range)	55.1 (16.2–167.5)	26.7 (6.4–111.9)	0.357
PTC (%)	10 (62.5)	6 (37.5)	0.751
US echogenicity—hyper/iso/hypo (%)^b^	3/5/0 (21.43/35.71/0)	0/2/4 (0/14.29/28.57)	**0.019**
Microcarcinoma (%)	3 (18.75)	1 (6.25)	1
Histopathological variant—classical/follicular (%)	4/6 (25/37.5)	4/2 (25/12.5)	0.608
Multifocal (%)	4 (25)	2 (12.5)	1
Time of followup—yrs (range)	9 (3–18)	8.5 (3–14)	0.785
Smoking (%)	4 (25)	1 (6.25)	0.588
Time of treatment—yrs (range)	3.5 (1–6)	7 (2–14)	0.062
Number of nodules (range)	1.5 (0–5)	3 (1–5)	0.148
Size of largest nodule on US—cm (range)	2.2 (0.5–6.8)	2.1 (1–4.3)	0.796

^a^ *n* = 57.

^b^ *n* = 14.

## References

[1] Holm LE, Blomgren H, Löwhagen T (1985). Cancer risks in patients with chronic lymphocytic thyroiditis. *The New England Journal of Medicine*.

[2] Pacini F, Elisei R, Di Coscio GC (1988). Thyroid carcinoma in thyrotoxic patients treated by surgery. *The Journal of Endocrinological Investigation*.

[3] Cantalamessa L, Baldini M, Orsatti A, Meroni L, Amodei V, Castagnone D (1999). Thyroid nodules in graves disease and the risk of thyroid carcinoma. *Archives of Internal Medicine*.

[4] Pazaitou-Panayiotou K, Michalakis K, Paschke R (2012). Thyroid cancer in patients with hyperthyroidism. *Hormone and Metabolic Research*.

[5] Yu GP, Li JCL, Branovan D, McCormick S, Schantz SP (2010). Thyroid cancer incidence and survival in the national cancer institute surveillance, epidemiology, and end results race/ethnicity groups. *Thyroid*.

[6] Belfiore A, Garofalo MR, Giuffrida D (1990). Increased aggressiveness of thyroid cancer in patients with Graves’ disease. *Journal of Clinical Endocrinology and Metabolism*.

[7] Holm LE, Blomgren H, Lowhagen T (1985). Cancer risks in patients with chronic lymphocytic thyroiditis. *The New England Journal of Medicine*.

[8] Singh B, Shaha AR, Trivedi H, Carew JF, Poluri A, Shah JP (1999). Coexistent Hashimoto’s thyroiditis with papillary thyroid carcinoma: impact on presentation, management, and outcome. *Surgery*.

[9] Shih ML, Lee JA, Hsieh CB (2008). Thyroidectomy for Hashimoto’s thyroiditis: complications and associated cancers. *Thyroid*.

[10] Shabana W, Peeters E, De Maeseneer M (2006). Measuring thyroid gland volume: should we change the correction factor?. *American Journal of Roentgenology*.

[11] Dobyns BM, Sheline GE, Workman JB (1974). Malignant and benign neoplasms of the thyroid in patients treated for hyperthyroidism: a report of the cooperative thyrotoxicosis therapy. Follow up study. *Journal of Clinical Endocrinology and Metabolism*.

[12] Belfiore A, Russo D, Vigneri R (2001). Graves' disease, thyroid, nodules and thyroid cancer. *Clinical Endocrinology*.

[13] Kim WB, Han SM, Kim TY (2004). Ultrasonographic screening for detection of thyroid cancer in patients with Grave’s disease. *Clinical Endocrinology*.

[14] Filetti S, Belfiore A, Amir SM (1988). The role of thyroid-stimulating antibodies of Graves’ disease in differentiated thyroid cancer. *The New England Journal of Medicine*.

[15] Mazzaferri EL (1993). Management of a solitary thyroid nodule. *The New England Journal of Medicine*.

[16] Kim WB, Han SM, Kim TY (2004). Ultrassonographic screening for detection of thyroid cancer in patients with Graves' disease. *Clinical Endocrinology*.

[17] Rago T, Fiore E, Scutari M (2010). Male sex, single nodularity, and young age are associated with the risk of finding a papillary thyroid cancer on fine-needle aspiration cytology in a large series of patients with nodular thyroid disease. *European Journal of Endocrinology*.

[18] Giles YS, Fatih T, Harika B, Yersu K, Tarik T, Serdar T (2008). The risk factors for malignancy in surgically treated patients for Graves’ disease, toxic multinodular goiter, and toxic adenoma. *Surgery*.

[19] Carnell NE, Valente WA (1998). Thyroid nodules in Graves’ disease: classification, characterization, and response to treatment. *Thyroid*.

[20] Erbil Y, Barbaros U, Ozbey N (2008). Graves' disease, with and without nodules, and the risk of thyroid carcinoma. *The Journal of Laryngology & Otology*.

[21] Anderson L, Middleton WD, Teefey SA (2010). Hashimoto thyroiditis: part 2, sonographic analysis of benign and malignant nodules in patients with diffuse Hashimoto thyroiditis. *American Journal of Roentgenology*.

[22] Gul K, Dirikoc A, Kiyak G (2010). The association between thyroid carcinoma and Hashimoto’s thyroiditis: the ultrasonographic and histopathologic characteristics of malignant nodules. *Thyroid*.

[23] Takashima S, Matsuzuka F, Nagareda T, Tomiyama N, Kozuka T (1992). Thyroid nodules associated with Hashimoto thyroiditis: assessment with US. *Radiology*.

[24] Solivetti FM, Bacaro D, Cecconi P, Baldelli R, Marandino F (2004). Small hyperechogenic nodules in thyroiditis: usefulness of cytological characterization. *Journal of Experimental and Clinical Cancer Research*.

[25] Anil C, Goksel S, Gursoy A (2010). Hashimoto’s thyroiditis is not associated with increased risk of thyroid cancer in patients with thyroid nodules: a single-center prospective study. *Thyroid*.

